# Inhibition of Methicillin-resistant *Staphylococcus aureus*-induced cytokines mRNA production in human bone marrow derived mesenchymal stem cells by 1,25-dihydroxyvitamin D_3_

**DOI:** 10.1186/1471-2121-15-11

**Published:** 2014-03-25

**Authors:** Aparna Maiti, William A Jiranek

**Affiliations:** 1 Department of Orthopaedic Surgery, Orthopaedic Research Laboratory, 1112 East Clay Street, Richmond, USA; 2 Department of Orthopaedic Surgery, Virginia Commonwealth University School of Medicine, 1250 East Marshall Street, Richmond, VA 23298-0614, USA

**Keywords:** Toll like receptors (TLRs), Methicillin-resistant Staphylococcus aureus (MRSA), Human Mesenchymal Stem Cells (hMSCs), Osteogenesis, 1,25(OH)_2_D_3_ (Vitamin D), Interleukin 8 (IL-8), Infection, Chronic inflammation, Histone methylation

## Abstract

**Background:**

Methicillin-resistant *Staphylococcus aureus* (MRSA) is the predominant cause of bone infection. Toll like receptors (TLRs) are an important segments of host response to infection and are expressed by a variety of cells including human mesenchymal stem cells (hMSCs). The active form of Vitamin D, 1,25-dihydroxyvitamin D3 (1,25(OH)_2_D_3_) has potent immunoregulatory properties, but the mechanism remains poorly understood. The genomic action of 1,25(OH)_2_D_3_ is mediated by vitamin D receptor (VDR), hormone-regulated transcription factor. VDR interacts with co-activators and co-repressors are associated with chromatin histone modifications and transcriptional regulation. The aim of our study is to explore MRSA-induced TLRs-mediated pro-inflammatory cytokines expression in hMSCs. Further, we hypothesized that 1,25(OH)_2_D_3_ inhibits MRSA-induced cytokines synthesis in hMSCs via inhibition of NF-кB transcription factor. Finally, we explored the regulatory role of 1,25(OH)_2_D_3_ in MRSA-mediated global epigenetic histone H3 mark, such as, trimethylated histone H3 lysine 9 (H3K9me3), which is linked to gene silencing.

**Results:**

Quantitative PCR data revealed that MRSA-infection predominantly induced expression of TLRs 1, 2, 6, NR4A2, and inflammatory cytokines IL-8, IL-6, TNFα in hMSCs. MRSA-mediated TLR ligands reduced osteoblast differentiation and increased hMSCs proliferation, indicating the disrupted multipotency function of hMSCs. Pretreatment of 1,25(OH)_2_D_3_ followed by MRSA co-culture inhibited nuclear translocation of NF-кB-p65, reduced expression of NR4A2 and pro-inflammatory cytokines IL-8, IL-6, and TNFα in hMSCs. Further, NF-κB-p65, VDR, and NR4A2 were present in the same nuclear protein complex, indicating that VDR is an active part of the nuclear protein complexes for transcriptional regulation. Finally, 1,25(OH)_2_D_3_ activated VDR, restores the global level of H3K9me3, to repress MRSA-stimulated inflammatory cytokine IL-8 expression. Pretreatment of 5-dAZA, DNA methylatransferases (Dnmts) inhibitor, dramatically re-expresses 1,25(OH)_2_D_3_-MRSA-mediated silenced IL-8 gene.

**Conclusions:**

This data indicates that TLR 1, 2, and 6 can be used as markers for localized *S. aureus* bone infection. 1,25(OH)_2_D_3_-VDR may exhibits its anti-inflammatory properties in MRSA-stimulated infection by inhibiting nuclear translocation of NF-kB-p65 and transcripts of IL-8, IL-6, TNFα, and NR4A2 in hMSCs. Finally, 1,25(OH)_2_D_3_-activated VDR, acting as an epigenetic regulator, inhibits synthesis of cytokines in MRSA-stimulated infection by restoring the global level of H3K9me3, a histone H3 mark for gene silencing.

## Background

*Staphylococci*, in particular Methicillin-resistant *Staphylococcus aureus* (MRSA), are the predominant cause of bone and joint infection. These infections cause serious morbidity and are often difficult to treat [1]. Recent evidence demonstrates that bacterially infected osteoblasts secrete chemokines and cytokines, suggesting that these cells are playing important role in combating localized infection through inflammation [2,3]. The goal of this study was to evaluate MRSA infection mediated host responses, and the molecular mechanisms of host defenses. Human Bone marrow derived mesenchymal stem cells (hMSCs) are known to support hematopoiesis to regenerate bone, cartilage and adipose tissues. Thus hMSCs possess complex biological functions which maintain the microenvironment of bone marrow. In addition, hMSCs are well known for their immunoregulatory characteristics [4]. They express pattern recognition receptors (PRRs) which recognize pathogenic molecules. The PRRs found in hMSCs are Toll like receptors (TLRs) [4]. There are currently 11 known mammalian TLRs of which TLR1-10 are functional in humans [5]. TLRs are the family of conserved transmembrane receptors, which are expressed by a variety of immune cells, and non-immune cells including mesenchymal stem cells. TLRs recognize several conserved PRRs like triacylated lipoprotein (TLR1 and TLR2), diacylated lipoprotein (TLR2, TLR6), double stranded RNA (TLR3), lipopolysaccharide (TLR4), flagellin (TLR5), single stranded RNA (TLR8) and unmethylated DNA (TLR9) as well as viral and bacterial nucleic acids [6-8]. Activation of TLR by its ligand results in several biological outcomes ranging from secretion of cytokines, rapid cellular differentiation, apoptosis, and up regulation of antimicrobial activity [9-11]. Thus in the present study, we have used hMSCs as our cell model to survey the expression of all known TLRs in response to MRSA co-culture condition which increased inflammation. Activation of TLRs with MRSA ligands predominantly increased TLR 1, 2 and 6 expression including transcription factor NR4A2, and pro-inflammatory cytokines e.g. IL-8, IL-6 and TNFα that altered normal function of hMSCs cell biology.

The active form of vitamin D, 1,25(OH)_2_D_3,_ the secosteroid hormone that is well known for calcium homeostasis. The genomic action of this hormone is mediated by nuclear receptor-vitamin D receptor (VDR). 1,25(OH)_2_D_3_ deficiency causes rickets in children and osteomalacia in adults. Low 1,25(OH)_2_D_3_ is also linked to increased disease activity in rheumatoid arthritis, and increased susceptibility to bacterial infection [12-14]. In addition TLR activation in human monocytes results in expression of the vitamin D receptor and vitamin D hydroxylase (cyp27B1) and antimicrobial peptide cathelicidin expression that has direct antimicrobial activity against *M. tuberculosis* when co-incubated with the bacteria [15]. Like other nuclear receptors, VDR interacts with co-activators and co-repressors during the transcriptional cycle, and these interactions combine to determine histone modifications [16-18]. Thus it is well established that 1,25(OH)_2_D_3_ is an important modulator of immune system. However, the precise molecular mechanisms that are regulated by VDR following TLR induction are not well defined. In the present study, we sought to examine the ability of 1,25(OH)_2_D_3_ to modulate the MRSA mediated inflammatory response in hMSCs. 1,25(OH)_2_D_3_ treatment followed by MRSA co-culture inhibit nuclear translocation of NF-кB-p65 and reduced cytokines (IL-6, IL-8, and TNFα) and NR4A2 expressions in hMSCs. Finally, 1,25(OH)_2_D_3_ activated VDR restores the global level of H3K9me3 to repress MRSA-stimulated inflammatory cytokine IL-8 expression. In addition, 5-dAZA, an inhibitor of DNA methylatransferases, reduces DNA and histone methylation, enhances IL-8 gene expression, furthermore, it dramatically re-expresses the 1,25(OH)_2_D_3_-MRSA-mediated silenced IL-8 gene.

## Results

### Methicillin-resistant *Staphylococcus aureus* (MRSA)-infection predominantly induces TLRs 1, 2, and 6 expressions in hMSCs

**Figure 1:**
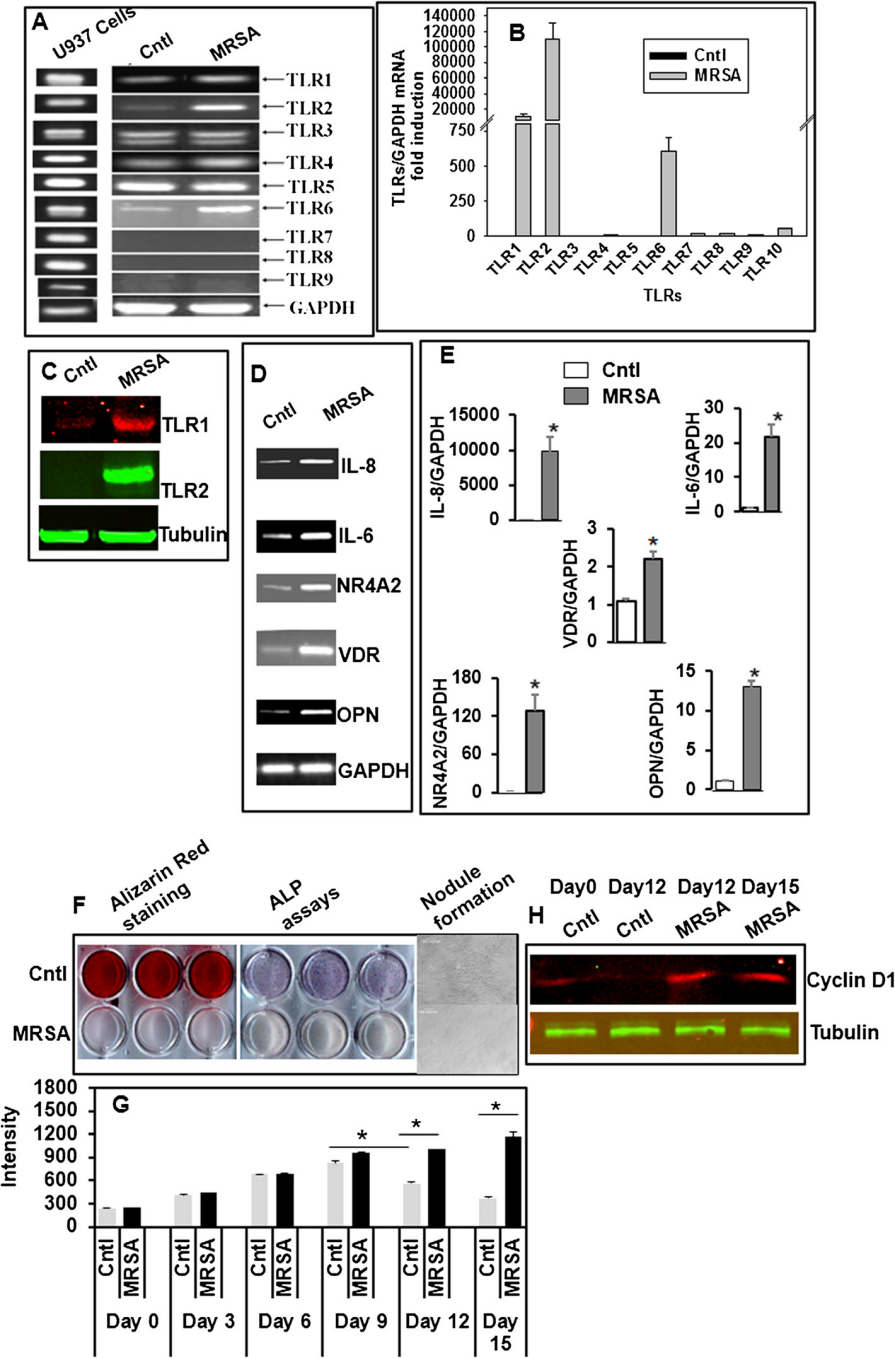
**MRSA co-culture induces TLRs expression by hMSCs.** Human MSCs total RNA was subjected to RT-PCR and qRT-PCR using SYBR green assay with TLRs specific primes. Representative RT-PCR of each TLR was shown. Human MSCs with no bacterial infection cDNA was used as control. U937 cell line cDNA was used as positive control for PCR amplification **(A)**. GAPDH was used as a reference. The relative mRNA expression was determined by the delta-delta comparative threshold (ddCT) method. **(B)**. TLR1 and TLR2 protein expression in MSCs are shown **(C)**. Cytokines stimulation in hMSCs by MRSA. RT-PCR was done with human specific primers of IL-8, IL-6, NR4A2, VDR, osteopontin (OPN), and GAPDH as a control **(D)**. RT-PCR data in hMSCs were further confirmed with qRT-PCR analysis using SYBR green assay, where GAPDH as an endogenous control **(E)**. Data are from n = 3 independent experiments done in triplicate. *P < 0.05 compared to control. MSCs were co-culture with and without *S.aureus* and cultured in osteogenic medium supplemented with dexamethasone (0.1μM). Alizarin Red staining was done at day 15. Alkaline phosphatase activity was measured at day 9 from cell lysate as released p-nitrophenol. At day 12, cells showed change in morphology and bone nodule formation in cultures of differentiating osteoblasts. Representative picture was shown. Original magnifications are 10× **(F)**. Cells were incubated with DRAQ5 for 5 min followed by washing with PBS and scanned in Li-Cor Odyssey Infrared Scanner. Equal area scanned intensities were plotted against samples of different day of treatment **(G)**. n = 3, * P < 0.002 compared to control. Western blot analysis of Cyclin D1 using Day12 and Day15 MRSA co-culture hMSCs protein extracts compared to Day 0, and Day 12 control extracts **(H)**.

In order to understand the differential TLRs members activated in hMSCs due to MRSA infection, we treated *in vitro* cultured adherent hMSCs with live MRSA as described in Materials and Methods. The TLRs mRNA expression pattern was analyzed by semi quantitative RT-PCR, using validated human specific primers of TLR1-9 in response to co-cultured with or without MRSA in hMSCs. We used *in vitro* cultured hMSCs without any bacterial infection as our control treatment. Three independent PCR run were done with hMSCs isolated from three independent donors to check consistency of the TLR expression in control cells, before doing MRSA co-culture as treatment. As shown in Figure 1, the expression levels of TLR3, TLR4 and TLR5 mRNAs in control hMSCs (without co-culture with live MRSA) were consistently high. By contrast, low expression level of TLR1, TLR2 and TLR6 and no expression of TLR 7, TLR 8, and TLR 9 were detected in control hMSCs, similar with the results obtained by Liotta et al. [19]. By contrast, we found that co-culture with live MRSA increased expression level of TLR1, TLR2, and TLR6 (Figure 1A). We used U937 human macrophage cells as positive control for human TLRs mRNA expressions with the same set of primers. The semi quantitative RT-PCR results of TLR mRNA expressions (Figure 1A) were further confirmed by quantitative real time PCR (qPCR) (Figure 1B). Furthermore, MRSA treatment in hMSCs also induced TLR proteins as confirmed western blotting (Figure 1C). When TLRs are activated by its ligands in hMSCs, it leads to activation of several known inflammatory pathways, synthesize and secrete cytokines, chemokines, and growth factors. This activation can lead to changes of basic function of bone marrow derived mesenchymal stem cells [4]. In immune cells, TLR activation signal leads to secretion of a variety of pro-inflammatory cytokines [20,21]. We therefore hypothesized that MRSA mediated TLRs activation up regulate several inflammatory genes. We analyzed mRNA expression of several cytokines by qPCR analysis from unstimulated or MRSA stimulated hMSCs. In agreement with NF-κB activation in response to co-culture with MRSA in MSCs, NF-κB-target pro-inflammatory cytokines IL-6, IL-8, and TNFα were significantly increased at the transcription level (Figure 1D-E), supporting similar data obtained by Lee et al. [20]. Figure 1D indicates that NR4A2 transcript is up regulated with 24 hrs of MRSA infection in hMSCs. This supports the clinical study that increased IL-8 and NR4A2 expression is also associated with the development and clinical symptoms of human inflammatory arthritis [22]. It was reported earlier that NR4A2 regulates the expression of various osteoblastic differentiation markers including osteopontin (OPN), a glycoprotein detected in several tissues in the body [23]. Interestingly, MRSA as TLR ligands stimulates OPN transcripts while no change in collagen type I transcripts (data not shown) were observed in hMSCs (Figure 1D-E). VDR is a nuclear receptor that mediates most known functions of 1,25(OH)_2_D_3_, the active form of vitamin D [24]. VDR forms heterodimers with RXR once VDR is activated by 1,25(OH)_2_D_3_. VDR binds to the vitamin D response element in the target gene promoter to regulate gene transcription [24]. Interestingly, RT-PCR data indicated that MRSA up regulated VDR in hMSCs (Figure 1D). Semi quantitative RT-PCR data (Figure 1D) were further confirmed by qPCR SYBR green assay (Figure 1E). Altogether our data indicated that TLR activation by MRSA play important roles in pathogenesis of these bacteria in hMSCs. It was reported earlier that TLRs and their ligands control murine MSCs function [4,25]. To examine whether MRSA as TLR ligand has any effect on *in vitro* cultured hMSCs biology, we co-cultured freshly isolated hMSCs with and without MRSA and allowed to differentiate in osteogenic medium containing dexamethasone for 15 days. In Figure 1F, control cells (without MRSA co-culture) at day 12, strongly stimulated bone nodule formation with dexamethasone treatment as shown by microscopic representative image (magnification 10×). However, the presence of MRSA totally abolished hMSCs matrix calcification as measured by alizarin red staining and alkaline phosphatase (an osteogenic marker) assay. No nodule formation was observed in MRSA co-cultured hMSCs (Figure 1F). Cell proliferation assay was done at every 3 days interval, with DRAQ5, a fluorescent dye that stain DNA inside the cells (Figure 1G). It was observed that, control cells stopped proliferating and started forming nodule after day 9, however, MRSA co-culture enhanced proliferation in hMSCs and failed to differentiate into osteoblasts until day 15 (Figure 1G). As expected, expression of cells proliferation marker cyclin D1 remains elevated until 12-15 days of MRSA infection in hMSCs compared to control as shown by western blot analysis (Figure 1H). Thus, our data indicate the incomplete differentiation potential of hMSCs due to extracellular *S. aureus* infection.

### MRSA-induced nuclear NF-κB activity is inhibited by 1,25(OH)_2_D_3_ in hMSCs

**Figure 2:**
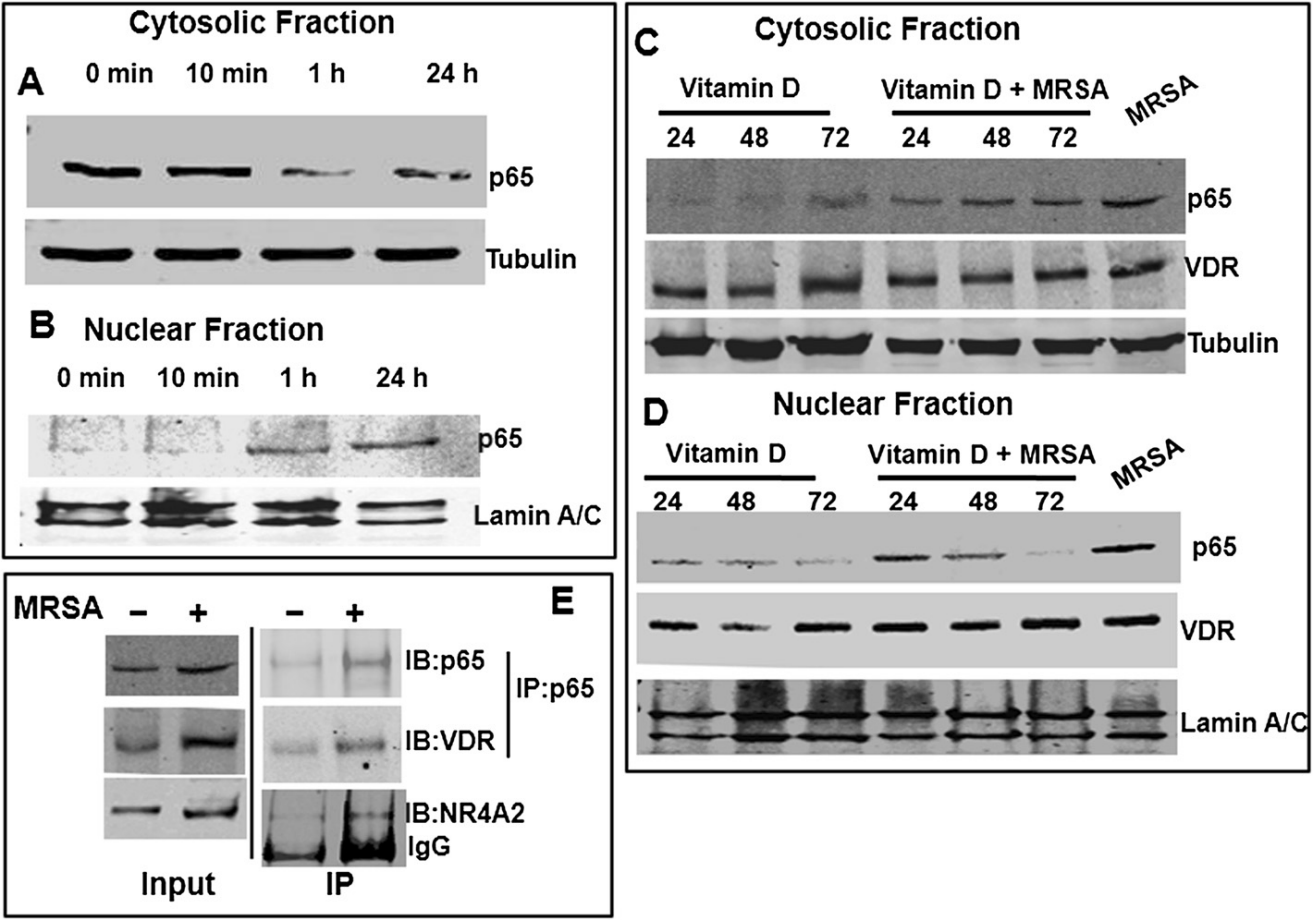
**1,25(OH)2D3 blocks NF-κB signaling in MRSA-stimulated hMSCs.** Human MSCs were co-culture with methicillin-resistant *Staphylococcus aureus* (MRSA) for 0 min, 10 min, 1 hr and 24 hrs and harvested for cytopalsmic and nuclear proteins extraction. Equal amount of proteins were used for western blot analyses with indicated antibodies **(A-B)**. Human MSCs were preincubated with vitamin D (100 nM) for 24, 48 and 72 hours, followed by methicillin-resistant *Staphylococcus aureus* (MRSA)-stimulation for 24 hours. Cytopalsmic and nuclear proteins were extracted for western blot analysis with indicated antibodies **(C-D)**. Experiments were done three times and representative blots were shown. NF-κB-p65 present in nuclear protein complexes with VDR, and NR4A2 of hMSCs. Human MSCs were stimulated with or without methicillin-resistant *Staphylococcus aureus* (MRSA) for 24 hrs and harvested for nuclear proteins extraction. Equal amount of proteins were used for immunoprecipitation (IP) with anti-NF-κB-p65 antibodies and immuno blots (IB) were done with indicated antibodies **(E)**. Experiments were done three times and representative blots were shown.

It has been shown that in immune cells, TLR activation is linked to activation of NF-кB dimer, a transcription factor consisting of p65 and p50 subunits, which enters the nucleus and regulates transcription of target genes [20,26,27]. In order to investigate whether TLRs expressed on hMSCs are functional, the outcome of TLR agonists-mediated TLR activation was performed using NF-κB nuclear localization by western blot as a read-out of its activation. Human MSCs were stimulated with MRSA as TLR ligands for increasing time points and subsequent nuclear and cytosolic fractionations studies were performed for western blot analysis for NF-κB-p65. In un-stimulated cells without any MRSA stimulation, NF-κB-p65 was localized mainly in cytoplasm (Figure 2A). As shown in Figure 2A-B, co-culture with MRSA induced NF-κB-p65 translocation to nucleus from cytosol within an hour and remains elevated in nucleus until 24 h of infection in hMSCs. This data further supporting the notion of functional status of TLRs signals which activate NF-κB transcription factor in human bone marrow-derived MSCs. Reduced 1,25(OH)_2_D_3_ intake or 1,25(OH)_2_D_3_ deficiency linked to susceptibility to *S. aureus* infection. 1,25(OH)_2_D_3_ is also well established for inhibiting inflammatory signals by inhibiting NF-κB activity in adipocytes [28]. In Figure 1B we also observed that TLR activation by MRSA increased VDR transcript. So we further investigated the effect of 1,25(OH)_2_D_3_ on nuclear translocation of the p65 subunit of NF-κB by western blot analysis in hMSCs. Our data indicated that pretreatment of hMSCs with 100 nM of 1,25(OH)_2_D_3_ for 72 hrs followed by MRSA stimulation for 24 hrs almost completely blocked NF-κB-p65 nuclear translocation (Figure 2C-D). To determine whether NF-κB-p65, VDR and NR4A2 could bind to the MRSA stimulated transcription complex *in vivo*, we performed immunoprecipitation assay. Human MSCs were stimulated with or without MRSA for 24 hrs and nuclear protein was used to perform immunoprecipitation with NF-κB-p65 rabbit polyclonal antibody. Our data indicated that NF-κB-p65, VDR and NR4A2 were present in the same nuclear protein complex (Figure 2E), indicating that VDR is an active part of the nuclear protein complexes for transcriptional regulation [29].

### Inhibitory effect of 1,25(OH)_2_D_3_ on MRSA-stimulated inflammatory cytokines mRNA expressions in hMSCs

**Figure 3:**
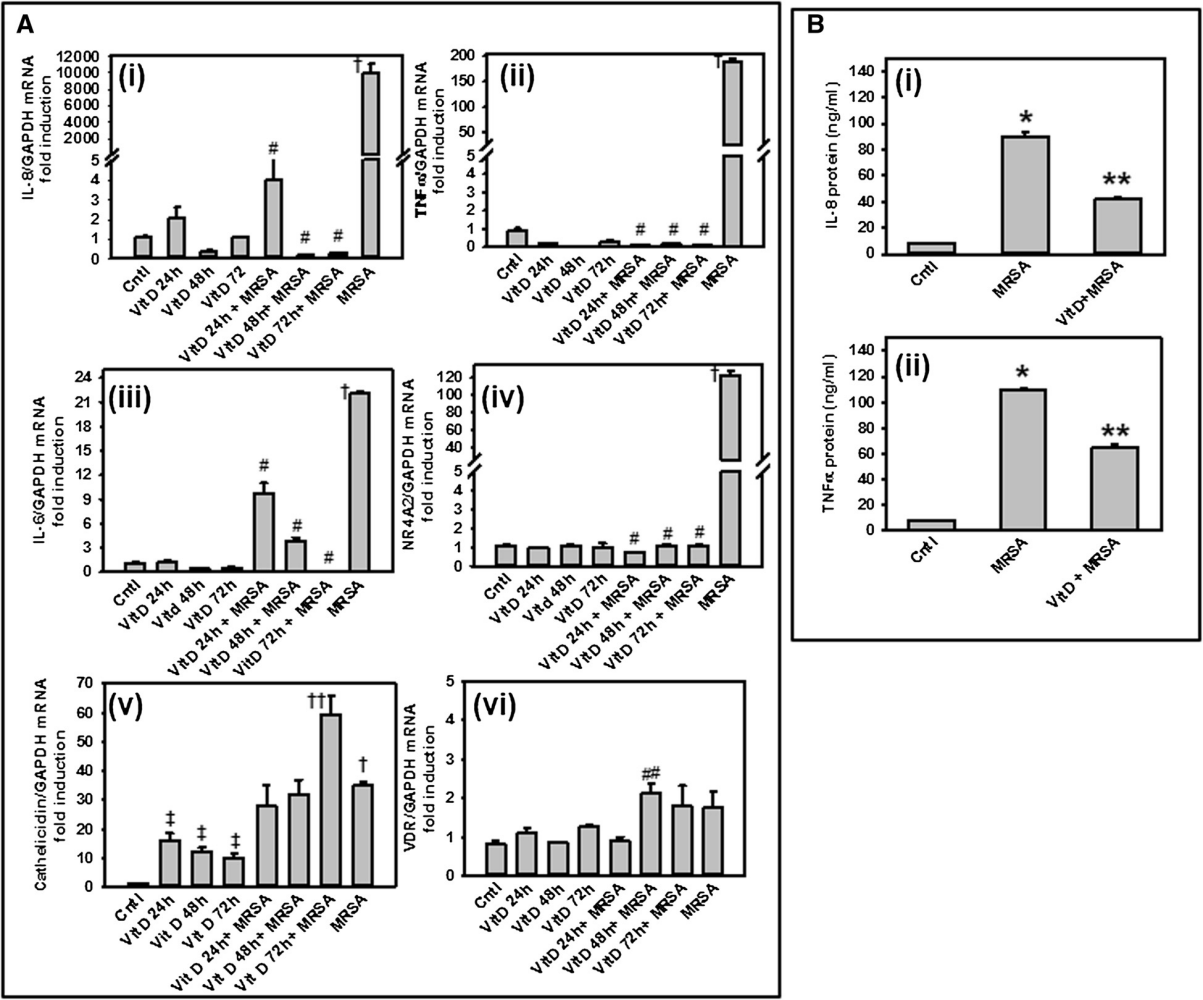
**Effect of 1,25(OH)**_**2**_**D3 pretreatment on MRSA-induced cytokines expressions of hMSCs. (A)** Human MSCs were preincubated with vitamin D (100 nM) for 24, 48 and 72 hours, followed by methicillin-resistant *Staphylococcus aureus* (MRSA)-stimulation for another 24 hours. Total RNA from hMSCs was isolated and processed for cDNA synthesis. Real-time quantitative PCR was performed with human specific primers of IL-8 **(A i)**, TNFα **(A ii)**, IL-6 **(A iii)**, NR4A2 **(A iv)**, Cathelicidin **(A v)**, and VDR **(A vi)**, using SYBR green assay. Endogenous control human GAPDH was used as a reference. The relative mRNA expression was determined by the delta-delta comparative threshold (ddCT) method. Data are from n = 3 independent experiments done in triplicate. † P < 0.05 compared to control. # P < 0.05 compared to MRSA. **††** P < 0.05 compared to MRSA. ‡ P < 0.05 compared to control. ##P < 0.05 compared to control. Effect of 1,25(OH)2D3 preincubation on MRSA-stimulated IL-8, and TNFα secretion **(B)**. Human MSCs were preincubated with 1,25(OH)2D3 (100 nM) for 48 hrs, followed by MRSA- stimulation for another 24 hrs. IL-8 **(B i)**, and TNFα **(B ii)** protein secretion were determined by ELISA in supernatants. Data are from n = 3 independent experiments done in triplicate. *P < 0.0001 for MRSA vs. Cntl stimulation, **P < 0.0003 for 1,25(OH)_2_D_3_ vs. MRSA stimulation.

Interestingly, earlier studies have shown that 1,25(OH)_2_D_3_ can suppress the release of TNF-α [30], the expression of IL-8 in human periodontal ligament cells stimulated with *Porphyromonas gingivalis,* associated with chronic periodontitis [31], LPS-stimulated IL-6 protein and mRNA synthesis in two human adipocyte models via interference with NF-κB signaling [28]. Moreover, 1,25(OH)_2_D_3_ effectively up-regulates the synthesis of the anti-inflammatory cytokine IL-10 and induces IL-10 receptor expression *in vitro*[32]. In contrast, 1,25(OH)_2_D_3_ deficiency increases TNF-α levels in healthy women [33]. In order to examine whether 1,25(OH)_2_D_3_ pretreatment down regulate MRSA-mediated inflammatory responses in hMSCs, we pretreated 100 nM of 1,25(OH)_2_D_3_ for 24, 48 and 72 hrs, followed by MRSA co-culture for another 24 hrs. Real-time quantitative PCR was performed with human specific primers of IL-8, IL-6, TNF-α, NR4A2, VDR, Cathelicidin using SYBR green assay. Our results showed that MRSA mediated inflammatory responses up regulated VDR expression (Figure 3A vi), furthermore, 1,25(OH)_2_D_3_ pretreatment inhibits MRSA-stimulated IL-8, IL-6 transcripts in a time dependent manner (Figure 3A i and 3A iii). TNF-α and NR4A2 transcripts are inhibited within 24 hrs of 1,25(OH)_2_D_3_ pretreatment (Figure 3A ii, 3A iv). Pretreatment with 1,25(OH)_2_D_3_ followed by MRSA co-culture caused time dependent increase of cathelicidin transcript (Figure 3A v). 1,25(OH)_2_D_3_ pretreatment to control hMSCs (without MRSA stimulation) has no effect on TNF-α and NR4A2 transcripts (Figure 3A ii, 3A iv). Taken together, 1,25(OH)_2_D_3_ was able to significantly reduce MRSA-induced IL-8, IL-6, TNF-α and NR4A2 mRNA expressions in hMSCs. This effect was observed both at concomitant incubation with MRSA and 1,25(OH)_2_D_3_ together (data not shown) and pretreatment with 1,25(OH)_2_D_3_ followed by MRSA co-culture in hMSCs. Furthermore, MRSA infection in hMSCs induced many pro-inflammatory cytokines protein expression, such as TNF-α, and IL-8, and their secretion into the culture medium (Figure 3B i-ii).

Interestingly, pretreatment with 1,25(OH)_2_D_3_ followed by MRSA co-culture in hMSCs significantly blocked secretion of IL-8, and TNF-α proteins (Figure 3i- 3ii).

### 1,25(OH)_2_D_3_ affect the global level of histone H3 lysine 9 trimethylation (H3K9me3) to repress MRSA-stimulated IL-8 transcription

**Figure 4:**
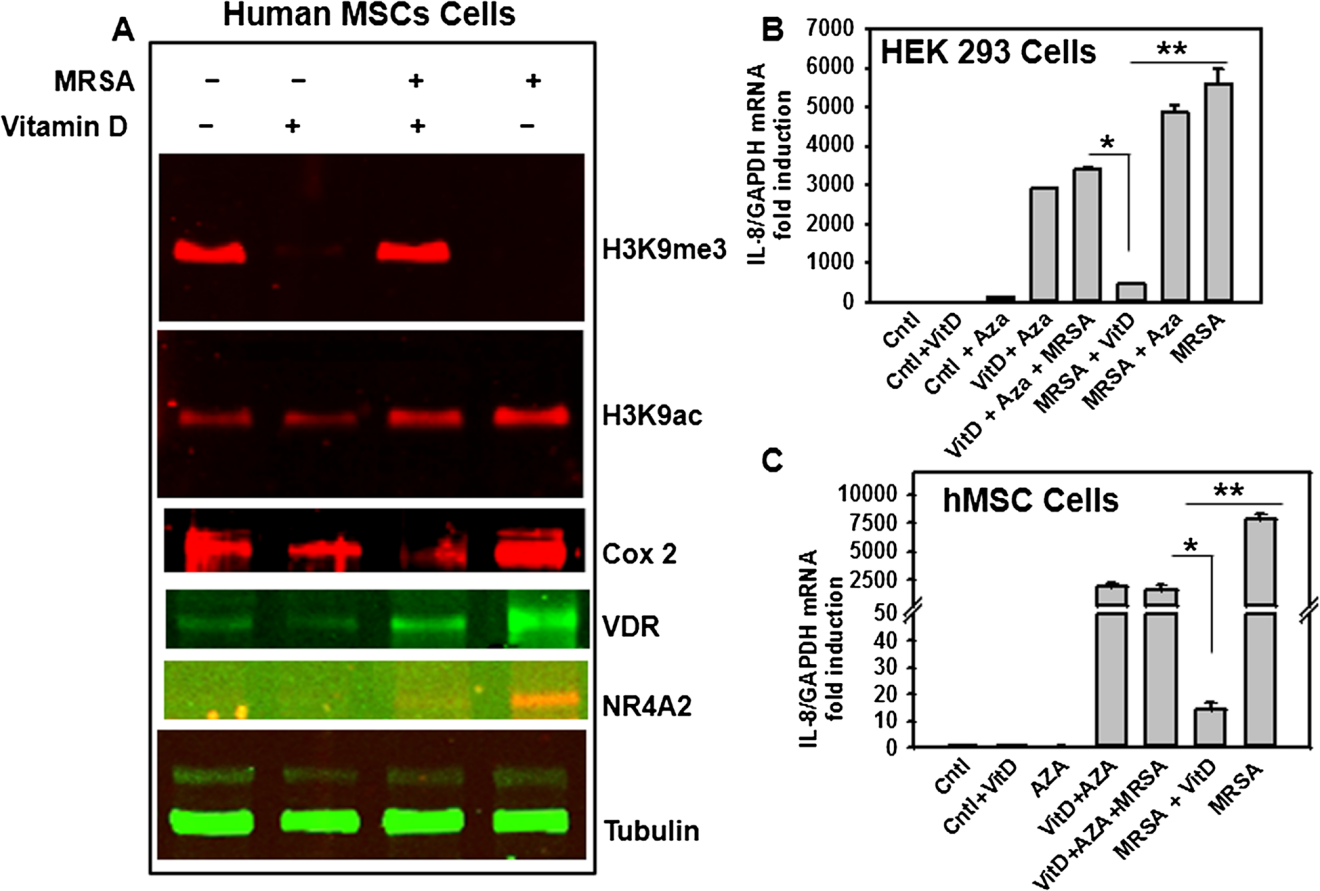
**1,25(OH)**_**2**_**D3 pretreatment increases global histone H3 methylation (H3K9me3) to reduce MRSA-mediated cytokines synthesis.** Human MSCs were pretreated 24 hrs with 1,25(OH)_2_D3 (100 nM) followed by co-culture with methicillin-resistant *Staphylococcus aureus* (MRSA) for another 24 hrs and harvested for total cellular proteins extraction. Equal amount of proteins were used for western blot analyses with indicated antibodies **(A)**. Experiments were done three times and representative blots were shown. HEK293 cells **(B)**, and human MSCs **(C)** were treated with or without 5Aza deoxycytidine (AZA) for 2 hrs followed by with or without 24hrs preincubated with 1,25(OH)_2_D_3_ (100 nM) followed by methicillin-resistant *Staphylococcus aureus* (MRSA)-stimulation for 24 hrs. Total RNA from hMSCs was isolated and processed for cDNA synthesis. Real-time quantitative PCR was performed with human specific primers of IL-8, using SYBR green assay. Endogenous control human GAPDH was used as a reference. The relative mRNA expression was determined by the delta-delta comparative threshold (ddCT) method. Data are from n = 3 independent experiments done in triplicate. *P < 0.02 for VitD + MRSA vs.VitD + AZA + MRSA stimulation, **P < 0.0002 for MRSA vs. VitD + MRSA stimulation.

1,25(OH)_2_D_3_, a hormonally active form of vitamin D3, is a repressive signal that binds to and activates the nuclear vitamin D receptor (VDR) [34,35]. A basic helix–loop–helix transcriptional activator (VDR interacting repressor, VDIR) regulates the transcription of CYP27B1 by the negative 1,25(OH)_2_D_3_ response element (nVDRE) [36]. Heterodimers of 1,25(OH)_2_D_3_-bound VDR and retinoid X receptor (RXR) repress the activation of VDIR that is bound upon the nVDRE by means of the histone deacetylase (HDAC) co-repressor complex [37]. The DNA methyltrasferase (Dnmt1) interacted with both VDR and VDIR in a ligand-dependent manner. Indeed, treatment with 1,25(OH)_2_D_3_ induced DNA methylation at CpG sites in the promoter and exon regions of the CYP27B1 gene [38]. It has been shown that H3K9 methylation promotes DNA methylation and serve as a critical mark for DNA methylation and gene silencing [39,40]. Methylation of lysines H3K9 and H3K27 are correlated with transcriptional repression [41]. Particularly, H3K9me3 is highly correlated with constitutive heterochromatin [42]. Angrisano et al. [43] reported that LPS induces early histone H3 methylation and acetylation changes at the promoter region of IL-8 gene in HT-29, a human intestinal epithelial cell line. Thus, 1,25(OH)_2_D_3_ may regulate expression of genes by epigenetic modifications of DNA and histones. In order to understand the molecular mechanisms how 1,25(OH)_2_D_3_ preincubation inhibiting inflammation, we tested effect of 1,25(OH)_2_D_3_, an active chromatin modulator, on the global level of histone H3 lysine 9 trimethylation (H3K9me3) in hMSCs, with or without MRSA-co-cultured conditions. Interestingly, we found that 1,25(OH)_2_D_3_ pretreatment alone or MRSA–stimulation alone remarkably reduces global level of H3K9me3 compared to the control in hMSCs (Figure 4A). Furthermore, 1,25(OH)_2_D_3_ pretreatment followed by MRSA-stimulation restored global level of H3K9me3 compared to the control in hMSCs (Figure 4A). Acetylation of lysine 9 on histone H3 (H3K9ac) has been linked to gene activation and active transcription [44]. We have also tested the global level of H3K9ac in this condition in hMSCs cell extracts. Interestingly, we found that 1,25(OH)_2_D_3_ pretreatment alone doesn’t affect the global level of H3K9ac, however, MRSA–stimulation alone remarkably induced global level of H3K9ac compared to the control hMSCs (Figure 4A). Further, 1,25(OH)_2_D_3_ pretreatment followed by MRSA-stimulation did not affect the global level of H3K9ac compared to the MRSA-stimulation alone in hMSCs (Figure 4A). Similar with earlier report [45], MRSA-stimulation showed dramatic induction of Cox-2 protein (Figure 4A), NR4A2, VDR (Figures 1D-E, 3A iv, 3A vi, and 4A) and 1,25(OH)_2_D_3_ pretreatment followed by MRSA-stimulation dramatically reduced Cox-2, NR4A2 protein levels in hMSCs (Figure 4A and Figure 3A iv).

5-deoxy-azacytidine (5-dAZA), an epigenetic drug that inhibits DNA methyltransferases (DNMTs) inhibits DNA methylation and often is used *in vitro* to induce the re-expression of genes putatively silenced by promoter methylation [46,47]. In order to test whether inhibiting methylation event with 5-dAZA, inhibits DNA methylation and histone methylation, as a result can re-express the 1,25(OH)_2_D_3_-MRSA-mediated silenced pro-inflammatory IL-8 gene. In order to demonstrate whether 1,25(OH)_2_D_3_-MRSA silencing cytokines expression is universal we have used human embryonic kidney cells, HEK293 cells for the D-AZA experiments. Interestingly, we found that in HEK293 cells, 5-dAZA treatment reduces DNA and histone methylation, enhances IL-8 gene expression, furthermore, it dramatically re-expresses the 1,25(OH)_2_D_3_-MRSA-mediated silenced IL-8 gene (Figure 4B i). Similar data also obtained in hMSCs (Figure 4C ii). Altogether, our data supporting the notion that 1,25(OH)_2_D_3_-activated VDR is an epigenetic regulator and may inhibits synthesis of cytokines in MRSA-stimulated infection by restoring the global level of H3K9me3, a histone H3 mark for gene silencing.

## Discussion

Human mesenchymal stem cells (hMSCs) are known to maintain regeneration of mesodermal tissue throughout the life time of an individual, which is dependent on the balance between the ability to differentiate into specialized cell types and self-renew. Excess self-renew and over differentiation may lead to mutation or tumorigenesis [25]. As hMSCs are also well known for their immunomodulatory functions, it is important to understand the factors playing role in the recognition of pathogens, which can lead to substantial changes in basic cellular functions. So far the only pathogen recognition receptors found in hMSCs are Toll like receptors (TLRs). Identification of TLR expression patterns which are associated with recognition of specific microbial molecules is critical to understand pathogen-host interactions. In our study, we used *Staphylococcus* (MRSA), a common pathogen of human body. *Staphylococcus* in particular; *Staphylococcus aureus* is the predominant cause of bone infections. Staphylococcal bone infections are thus increasing concern and understanding of the interaction of these pathogens with the bone is central to the development of the novel therapeutic strategies required to treat increasing antibiotic resistant and persistent infections. Our study revealed that expression levels of TLR3, TLR4 and TLR5 mRNAs in control hMSCs (without co-culture with live bacteria) were consistently high. By contrast, low expression level of TLR1, TLR2 and TLR6 and no expression of TLR 7, TLR 8, and TLR 9 were detected in control hMSCs, similar with the results obtained by Liotta et al. [19]. Further confirmation of TLR response with validated human specific qPCR primers of TLRs revealed that in response to co-culture with live MRSA increased mRNA expression of TLR 1, 2, and 6 in hMSCs. In addition to inflammatory responses, TLRs have been shown to directly regulate cell survival and cellular proliferation in a variety of biological settings [48]. The TLRs which are found to express in control cells, are may be involved for normal cell survival and proliferation. In agreement, as reported earlier by Bandow et al. [49], we have shown that activation of TLRs in response to MRSA co-culture completely suppressed osteoblast differentiation and increased cell proliferation. Increased IL-8 and NR4A2 are associated with inflammatory arthritis [22,50,51]. NR4A2 sub family has emerged as potential regulators of cytokines and growth factor action through regulating the inflammatory response underlying several diseases. NR4A2 is rapidly and potentially induced by a range of cytokines, suggesting that these receptors act as potential transcriptional mediators of inflammatory signals [22,51-53]. We have tested several inflammatory genes expression in MRSA-mediated infection condition in hMSCs. We found that when hMSCs were co-culture with MRSA resulted in up regulation of IL-8, IL-6, TNFα, NR4A2, VDR, and OPN transcripts. Osteopontin (OPN) is expressed by several tissues, regulated by several signaling pathways and transcription factors that are associated with cancer progression [54]. Within bone tissue OPN plays a key role in cell adhesion, migration and survival. As IL-8 is the target gene for NR4A2, our data support the notion that NR4A2 could be a critical regulator of those gene involved in proliferation in infection-mediated TLR-activated hMSCs. Interestingly, we found that VDR expression is very high after treating hMSCs with MRSA. In our study we observed that hMSCs without any MRSA infection form distinct nodules and exhibited ALP activity and *de novo* formation of extracellular calcium deposition (Figure 1F), which is consistent with the previous observations found in control murine MSCs [25,55]. However, MRSA infection fully blocked extracellular calcium deposition and ALP staining (Figure 1F-G), while it promotes hMSCs proliferation (Figure 1G-H). This indicated clearly a shift of hMSCs to self-renewal rather differentiation due to extracellular MRSA infection.

In immune cells, TLRs ligands activate downstream signaling and translocate NF-κB-p65, a key transcription factor, to the nucleus for synthesis of its target genes include inflammatory cytokines IL-8, IL-6, and TNFα [26]. Recently, it has been implicated in human inflammatory joint disease; nuclear protein NR4A2 cooperatively interacted with NF-kB-p65 at the IL-8 promoter for its transcriptional activation with TNFα [22]. It has long been known that 1,25(OH)_2_D_3_ exert its anti-inflammatory effect by inhibiting NF-kB-p65 activity. Therefore, 1,25(OH)_2_D_3_ suppression of NF-κB activation has great biological and pathological relevance. However, it remains to be determined how vitamin D receptor (VDR) is directly involved in the regulation of the NF-κB pathway. Chen et al. [56], reported that VDR physically interact with IkB kinase to block NF-κB activation. In dendritic cells, 1,25(OH)_2_D_3_ inhibits IL-12 expression through targeting the NFκB pathway [57]; by directly suppresses RelB transcription [58]. In addition, it was reported that epigenetic regulation is essential to NF-kB-p65 transcriptional activity via lysine methylation [59,60]. We also found that pretreatment of hMSCs with 1,25(OH)2D3 followed by MRSA stimulation significantly blocked NF-κB-p65 nuclear translocation and inflammatory cytokines synthesis. Further, NF-κB-p65, VDR and NR4A2 were present in the same nuclear protein complex, indicating that VDR is an active part of the nuclear protein complexes for transcriptional regulation.

The actions of 1,25(OH)_2_D_3_ are mediated by the VDR, a transcription factor that belongs to nuclear hormone receptor superfamily [61]. Previous research showed that binding of 1,25(OH)_2_D_3_ to VDR in the cytoplasm of cells stimulates heterodimerization of VDR with RXR and the redistribution of the VDR-RXR-hormone complex to the nucleus [62-65,17,18]. Recently, it has been reported that VDR and many other nuclear receptors such as NR4A2 interacts with coactivators and corepressors during the transcriptional cycle [18]. Saijo et al. [66] has reported the involvement of NR4A2-CoREST corepressor complex signaling pathway in neuro-inflammation, which further explain the regulatory role of VDR-NF-kB-NR4A2 in inflammation.

The VDR, such as many other nuclear receptors, interacts with coactivators and corepressors during the transcriptional cycle [18,66] has reported recently, anti-inflammatory role of NR4A2-CoREST corepressor complex signaling pathway in neuro-inflammation, which further the regulatory role of VDR-NF-kB-NR4A2 in inflammation. Several related recent observations revealed that VDR-corepressor complexes could also repress transcription of genes in a ligand-dependent manner through epigenetic modification of DNA Methylation and histone methylation in the gene promoter regions [67-69]. Binding of NCOR1 at a specific VDR binding site was associated with the loss of H3K9ac and gain of H3K9me2 and H3K27me3 [18]. Methylation of lysines H3K9 and H3K27 is correlated with transcriptional repression [41], particularly; H3K9me3 is highly correlated with constitutive heterochromatin [42], associated with CpG methylation [70,71], a histone mark for transcription repression. Given that H3K9me levels can attract the machinery that drives DNA CpG methylation, we test whether inhibiting methylation event with 5-dAZA, inhibits DNA methylation and histone methylation can re-express the 1,25(OH)_2_D_3_-MRSA-mediated silenced inflammatory gene. Our data indicated for the first time that 1,25(OH)_2_D_3_ pre-treatment followed by MRSA treatment restored H3K9me3 levels, and blocked synthesis of cytokines. In addition, 5-dAZA treatment reduces DNA and histone methylation, enhances IL-8 gene expression, furthermore, it dramatically re-expresses the 1,25(OH)_2_D_3_-MRSA-mediated silenced IL-8 gene (Figure 4). Our study revealed an interrelated set of factors that explain the epigenetic mechanism of ligand bound VDR to inhibit the synthesis of cytokines. It is tempted to speculate that 1,25(OH)_2_D_3_-VDR-NR4A2 mediated global epigenetic mechanisms may directly regulate NF-kB-p65 translocation to the nucleus and epigenetically modulate targeted cytokine promoters for transcriptional inhibition. However further confirmation is required by detail studies whether VDR mediated epigenetic modification is on global transcription factors on cytokine promoters or NF-κB-p65 chaperoned by VDR and NR4A2 recruitment to the targeted gene promoters.

## Conclusions

Altogether, our data explain that TLR 1, 2, and 6 can be used as markers for localized *Staphylococcus* bone infection and supporting the notion that 1,25(OH)_2_D_3_ has potential role in inhibition of methicillin-resistant *Staphylococcus aureus* (MRSA)-stimulated infection mediated inflammation by inhibiting IL-8, IL-6, TNF-α and NR4A2 transcripts in hMSCs. 1,25(OH)_2_D_3_ inhibits translocation of NF-kB-p65, an important transcription factor for inflammation, to the nucleus, indicating its anti-inflammatory role in hMSCs. Further, we also partly demonstrated that 1,25(OH)_2_D_3−_VDR is an epigenetic regulator and may exhibit its anti-inflammatory properties by restoring transcription repression histone methylation mark (such as H3K9me3). 1,25(OH)_2_D_3_ may inhibit synthesis of cytokines by restoring histone methylation at the gene promoters. Further studies are required to elucidate the mechanism of 1,25(OH)_2_D_3_-activated VDR epigenetic mechanism inhibited synthesis of cytokines in MRSA-stimulated infection by restoring the global level of H3K9me3, a histone H3 mark for gene silencing.

## Methods

### Bone marrow aspiration and mesenchymal stem cells (hMSCs) isolation and culture

Fresh bone marrow was obtained by aspiration (5-10 ml aspirate) from proximal femur of patients undergoing primary hip arthroplasty. The mean age was 55 y (range 50-70 y). The study protocol was approved by the Ethics Committee, Institutional Review Board (IRB) of Virginia Commonwealth University, Medical Center, Richmond, Virginia, USA. Human Bone marrow derived Mesenchymal stem cells (hMSCs) were isolated from adult human bone marrow samples by the adherence method [72]. Briefly, the mononuclear cell fraction was isolated from red blood cells by density gradient centrifugation over histopaque 1077 (Sigma-Aldrich, St Louis MO). Isolated mononuclear cells were added to a T75 tissue culture flask containing DMEM high glucose medium supplemented with 10% FBS, 1% antibiotics (penicillin and streptomycin) and 2 ng/ml basic-FGF. Non-adherent cells were removed after 4 days and homogenous adherent cells (hMSCs) were expanded in the same medium.

### *In vitro* co-culture of hMSCs and *S. aureus* (MRSA)

Adherent hMSC cells were washed twice in PBS and resuspended in antibiotic-free medium (DMEM + 10% FBS + 2 ng/ml basic-FGF) at a density of 75,000 cell/ml and plated in 6-well tissue Plates. *S. aureus* (MRSA) (strain 2884D) (kindly gifted by Sam Boundy, Ph.D, Department of Internal Medicine, Division of Infectious Diseases, Virginia Commonwealth University School of Medicine) were grown overnight in Tryptocase Soy Broth (TSB) (Sigma, USA) in shaking conditions at 37°C until they reached an optical density of 1.0, pelleted cells washed twice in PBS and added to cells at a multiplicity of infection (MOI) of 1:100 for a set time period mentioned in each figures [73]. The MSCs were then washed with sterile PBS and used for proteins and RNA isolation.

### RNA extraction and real-time quantitative PCR

Human MSCs total RNA was isolated using Trizol reagent (Invitrogen Life Technologies, USA). Contamination of DNA was removed using DNase I treatment (Promega, USA). Equal amount of RNA (1 μg) was used for cDNA synthesis using the iSCRIPT cDNA synthesis kit (Bio-Rad, USA). All PCR products were visualized by ethidium bromide after 1% agarose gel electrophoresis. Human specific RT-PCR primers sequences of TLRs 1-9, IL-6, IL-8, NR4A2, VDR, and OPN, GAPDH as an endogenous housekeeping gene control used for this study were described before [22,74-76].

All semi quantitative RT-PCR data including TLRs expression in MSCs were again confirmed with human specific primers for qPCR using SYBR green assay. All the qPCR primers including human TLRs primers were obtained from online PrimerBank public resources (http://pga.mgh.harvard.edu/primerbank/index.html). The sequences of the qPCR primers were: hTLR1: (5′→3′) Forward TGAACCTCAAGCACTTGGACC and Reverse CCCATAA GTCTCTCCTAAGACCA; hTLR2: (5′→3′) Forward TCCAGCA CACGAA TACA CAGT and Reverse AGGCATCTGGTAGAGTCATCAA; hTLR3: (5′→3′) Forward TTGCCTTGTATCTACTTTTGGGG and Reverse TCAACACTGTTATGTTT GTGGGT; hTLR4: (5′→3′) Forward GTACCTGGGGAACAACCTCTT and Reverse GCAGCTTGACTAGACTCTCCA; hTLR5: (5′→3′) Forward TCCCTGA ACTCACGAGTCTTT and Reverse GGTTGTCAAGTCCGTAAAATGC; hTLR6: (5′→3′) Forward TTCTCCGACGGAAATGAATTTGC and Reverse CAGC GGTA GGTCTTTTGGAAC; hTLR7: (5′→3′) Forward TCCTTGGGGCTAG ATGG TTTC and Reverse TCCACGATCACATGGTTCTTTG; TLR8: (5′→3′) Forward TGTGAG TTATGCGCCGAAGAA and Reverse GTTTGGGGAACTTCCTGTAGTC; hTLR9: (5′→3′) Forward CTGCCACATGACCATCGAG Reverse GGACAGG GATAT GAGGGATTTGG; hTLR10: (5′→3′) Forward GATTTACTCTGGGACGACCTTTT and Reverse GTCAAGATAAGCCTTACCACCAA; hNR4A2: (5′→3′) Forward AGAGCTACAGTTACCACTCTTCG and Reverse GAGGTCCATGCTAA ACTTGACAA; hVDR: (5′→3′) Forward TCTCCAATCTGGATCTGAGTGAA and Reverse ACAGCTCTAGGGTCACAGAAG; hIL-8: (5′→3′) Forward AGG TGC AGT TTT GCC AAG GA and Reverse TTT CTG TGT TGG CGC AGT GT; hIL-6: (5′→3′) Forward GAG GCA CTG GCA GAA AAC AA and Reverse TGG CAT TTG TGG TTG GGT CA; hOPN: (5′→3′) Forward ATCCATGTGGTCATGGCTTT and Reverse GAAGGAGCTGAAGGAGCTGA; hTNF-α (5′→3′) Forward AGCCCATGTTGTAGCAAACC and Reverse TGAGGTACAGGCCCTCTGAT; hGAPDH: (5′→3′) Forward AAGGTGAAGGTCGGAGTCAAC and Reverse GGGGTCATTGATGGCAACAATA. Quantitative RT-PCR was performed using a *SYBR*® *Green* I kit (Bio-Rad, USA) in a CFX96 Real*-*Time PCR Detection System (Bio-Rad, USA) according to the manufacturer’s instructions. The following cycling parameters were used: 5 min at 94*°*C; 40 cycles of 45 s at 94*°*C, 45 s at 55*°*C and1min at 72*°*C; and finally 10 min at 72*°*C, followed by a final dissociation stage. Fold change of TLRs mRNA levels were calculated based on the threshold cycle (CT) values and normalized to levels of constitutively expressed housekeeping gene Glyceraldehyde 3-phosphate dehydrogenase (GAPDH) mRNA. The comparative CT method with the formula 2^−ΔΔCt^ was used to analyze the data. The formula used to calculate ΔCT = Avg *·* TLR CT *−* Avg *·* GAPDH CT. All reactions were replicated. Melting curve analysis showed accurate PCR performance with a single peak without any nonspecific products for both populations. We used in *vitro* cultured hMSCs without any bacterial inoculation as our control treatment. Three independent PCR runs were performed with MSCs isolated from three independent donors to check consistency of the TLR expression in control cells, before doing *S. aureus* infection as a treatment.

### Nuclear and cellular extract preparation, western blotting, and immunoprecipitation

Human MSC cells were rinsed twice in ice-cold PBS. Cell pellets were used to isolate nuclear and cytoplasmic fractions using NE-PER® Nuclear and Cytoplasmic Extraction kit (Pierce, USA). For other experiments, cell pellets were resuspended in the extraction buffer [10 mM TrisHCl, pH 7.5; 50 mM NaCl; 0.1 mM EDTA; 0.5% Triton X-100; 40 mM NaF; 1 mM β-glycophosphate; and 1:500 protease inhibitor cocktail (Sigma, USA)] and sonicated with the probe sonicator (Fisher, USA) setting at 3 for 10 seconds ×3 with 1 min interval on ice to make whole cell lysate. The cell lysates were centrifuged at 10,000 rpm for 10 min to obtain cell free extract for western blot analysis. Protein concentrations were measured (Bio-Rad protein assay) and stored at-80°C. Equal amounts of protein were separated by sodium dodecyl sulfate–polyacrylamide gel electrophoresis (SDS-PAGE), transferred to nitrocellulose, and immunoblotted with primary antibodies, anti-TLR1,2, Cyclin D1, Cox-2, Lamin A/C (Cell Signaling Technology, Inc. USA); H3K9me3, and H3K9ac (Abcam, USA); VDR, p65, NR4A2 (Santa Cruz Biotechnology, Inc. USA); Tubulin (Sigma, USA) and protein bands were detected using a Li-Cor Odyssey Infrared Scanner. For immunoprecipitation (IP) experiment, approximately 500 μg nuclear proteins were pre-cleaned with protein A/G beads (Santa Cruz Biotechnology, Inc. USA) for 1 h at 4°C with agitation with the IP buffer [10 mM TrisHCl, pH 7.5; 150 mM NaCl; 10% glycerol, 0.5% Triton X-100 (Sigma, USA)]. Protein A/G beads were pellet down at 3000 rpm for 4 min 4°C and discarded. Anti-p65 antibodies were used over night to immunoprecipitate p65. Finally, protein A/G beads were used to capture immune complexes. Beads were washed extensively with the IP buffer and processed for western blot analysis.

### Osteogenic differentiation of hMSCs

MSCs were continuously infected with live MRSA during osteogenesis to maintain the bacterial infected condition. Osteogenic differentiation of MSCs was performed in DMEM medium containing 10% FBS, 1% pens/strep antibiotics, 10 mM glycerol-2-phosphate disodium salt hydrate, 0.1 mg/ml ascorbic acid and 0.1 μM Dexamethasone (Dex) (Sigma-Aldrich). All experiments were done in triplicate in 6 well plates. Differentiation medium was replaced fresh at 3 day intervals from the start of the experiment.

### Alizarin red, alkaline phosphatase (ALP) and proliferation assay

For alizarin Red and Alkaline phosphatase assay at day 14 and day 9 respectively, monolayers of MSCs were fixed with 4% para-formaldehyde for 10 min at room temperature, followed by washes with PBS several times and then stained for Alizarin Red S (aqueous 2% Alizarin Red, pH 4.2; Sigma-Aldrich) or Alkaline phosphatase assay and washed several times with PBS to remove over staining. Alkaline phosphatase was stained from cell lysate as released p-nitrophenol from a Naphthol AS-MX phosphate substrate (Sigma- Aldrich) after 15 min of incubation at 37°C. Proliferation assay was done with 1 μM DRAQ5 [77] which stains DNA following manufacturer’s protocol and scanned in Li-Cor Odyssey Infrared Scanner. Equal area scanned intensities were plotted against samples of different day of treatment.

### Statistical analysis

Results are presented as means ± SE. For statistical analysis, Student’s unpaired t test for independent samples was used. A value of P < 0.05 was regarded as statistically significant. All analyses were performed using MS Office Excel 2013, and SigmaPlot12.0.

## Abbreviations

TLRs: Toll like receptors; MRSA: Methicillin-Resistant *Staphylococcus aureus*; hMSCs: human mesenchymal stem cells; 1,25(OH)2D3: 1,25-dihydroxyvitamin D_3_; VDR: vitamin D receptor; IL-6: Interleukin-6; IL-8: interleukin-8; TNFα: tumor necrosis α; HDAC: Histone deacetylase; DNMT: DNA methyltransferase; CpG: Cytosine-phosphate-guanine.

## Competing interests

The authors declare that they have no competing interests.

## Authors’ contributions

AM developed the concept, designed and carried out experiments. AM and WAJ analyzed data. AM created figures. AM, WAJ wrote the manuscript. Both authors read and approved the final manuscript.
